# Delayed Surgery after Neoadjuvant Treatment for Rectal Cancer Does Not Lead to Impaired Quality of Life, Worry for Cancer, or Regret

**DOI:** 10.3390/cancers13040742

**Published:** 2021-02-11

**Authors:** Vincent Maurice Meyer, Richtje R Meuzelaar, Yvonne Schoenaker, Jan-Willem de Groot, Edwin de Boer, Onno Reerink, Wouter de Vos tot Nederveen Cappel, Geerard L Beets, Henderik L van Westreenen

**Affiliations:** 1 Department of Surgery, Isala Hospitals, P.O. Box 10400, 8000 GK Zwolle, The Netherlands; r.r.meuzelaar@isala.nl (R.R.M.); i.j.h.schoenaker@isala.nl (Y.S.); h.l.van.westreenen@isala.nl (H.L.v.W.); 2 Department of Oncology, Isala Hospitals, P.O. Box 10400, 8000 GK Zwolle, The Netherlands; j.w.b.de.groot@isala.nl; 3 Department of Radiology, Isala Hospitals, 8025 AB Zwolle, The Netherlands; e.de.boer@isala.nl; 4 Department of Radiotherapy, Isala Hospitals, 8025 AB Zwolle, The Netherlands; o.reerink@isala.nl; 5 Department of Gastroenterology, Isala Hospitals, 8025 AB Zwolle, The Netherlands; w.h.de.vos@isala.nl; 6 Department of Surgery, Netherlands Cancer Institute, 1066 CX Amsterdam, The Netherlands; g.beets@nki.nl

**Keywords:** rectal cancer, watch and wait, neo-adjuvant treatment, non operative management

## Abstract

**Simple Summary:**

Rectal cancer patients with an initial (near) complete clinical response to neoadjuvant chemoradiotherapy can be repeatedly assessed to see if a complete response endures. Up to 75% of these patients are able to avoid surgery and its related complications. However, the remaining 25% who ‘fail’ will eventually have to undergo surgery. Although recent studies have shown that patients undergoing delayed surgery have promising surgical and oncological outcomes, it is not known how these patients fare in terms of quality of life. The aim of this study was to compare quality of life between these immediate and delayed surgery groups through validated questionnaires. Our study including 51 patients shows no difference in quality of life, worry for cancer, or decision regret. Therefore, from a quality of life perspective, this study supports a repeated response assessment strategy after chemoradiotherapy for rectal carcinoma to identify all complete responders.

**Abstract:**

Non operative management of complete clinical responders after neoadjuvant treatment for rectal cancer enjoys an increasing popularity because of the increased functional outcome results. Even a near complete response can evolve in a cCR, and therefore further delaying response assessment is accepted. However, up to 40% of patients will develop a regrowth and will eventually require delayed surgery. It is presently unknown if and to what extent quality of life of these patients is affected, compared to patients who undergo immediate surgery. Between January 2015-May 2020, 200 patients were treated with neoadjuvant therapy of whom 94 received TME surgery. Fifty-one (59%) of 87 alive patients returned the questionnaires: 33 patients who underwent immediate and 18 patients who underwent delayed surgery. Quality of life was measured through the QLQ-C30, QLQ-CR29, and Cancer Worry Scale questionnaires. Regret to participate in repeated response assessment protocol was assessed through the Decision Regret Scale. Exploratory factor analysis (EFA) and a ‘known groups comparison’ was performed to assess QLQ questionnaires validity in this sample. Higher mean physical function scores (89.2 vs. 77.6, *p* = 0.03) were observed in the immediate surgery group, which lost significance after correction for operation type (*p* = 0.25). Arousal for men was higher in the delayed surgery group (20.0 vs. 57.1, *p* = 0.02). There were no differences between surgical groups for the other questionnaire items. Worry for cancer was lower in the delayed surgery group (10.8 vs. 14.0, *p* = 0.21). Regret was very low (12–16%). EFA reproduced most QLQ C-30 and CR29 subscales with good internal consistency. Quality of life is not impaired in patients undergoing delayed TME surgery after neoadjuvant treatment for rectal cancer. Moreover, there is very low regret and no increase in worry for cancer. Therefore, from a quality of life perspective, this study supports a repeated response assessment strategy after CRTx for rectal carcinoma to identify all complete responders.

## 1. Introduction

Neoadjuvant chemoradiotherapy (CRTx) followed by total mesorectal excision (TME) surgery is the gold standard for locally advanced rectal carcinoma. A prognostic favorable subgroup of patients will develop a complete clinical response after CRTx. Increasing evidence shows that non-operative management (NOM) by a watch-and-wait (W&W) strategy leads up to 75% of patients avoiding surgery and its related complications, with excellent oncological results [1,2]. Therefore, W&W is gaining acceptance as an alternative to TME surgery.

However, it remains difficult to clinically identify a complete pathological response (pCR). Treatment related fibrosis and inflammation after CRTx impair the interpretation of digital rectal examination, MRI, endoscopy, and biopsies [3,4,5,6]. Response assessment is further complicated by the timing of examination. In a cohort of 49 patients with a near complete clinical response (near cCR) at 8–10 weeks, 90% (44) turned out to have a cCR at response assessment 6–12 weeks later. [7] True complete responders could even take 19 to 26 weeks to develop a cCR [8]. Therefore, repeated response assessment in good responders will lead to identification of more complete responders. However, no diagnostic test to detect a complete response is entirely accurate and some true complete responses will therefore not be recognized. Fortunately, almost all regrowths that occur in a W&W protocol are salvageable and oncological outcomes are promising [1,9]. In patients with a regrowth, even organ preservation remains possible [10]. Therefore, a repeated response assessment strategy in good responders with delayed or salvage surgery for those who ‘fail’ is a promising approach [1,2,11].

Several studies have shown that NOM leads to a higher health-related QoL compared to TME surgery [12,13]. Little is known about the quality of life of those patients who, after an initial W&W approach, eventually require TME surgery. QoL might be impaired because on top of the anticipatory distress, patients who actually develop a regrowth have to undergo the psychological distress of what they feared would happen, bringing extra feelings of uncertainty and fear of death [14,15]. While we likely benefit the good clinical responder group as a whole, do we ‘harm’ the patients that develop a regrowth from a QoL perspective? The goal of this study was to quantify the possible negative impact on quality of life and feelings of regret and worry for cancer in patients in a W&W program who eventually require TME surgery for a regrowth.

## 2. Materials & Methods

### 2.1. Study Design

This study is part of a multicenter prospective registration study “Wait-and-see” Policy for Complete Responders After Chemoradiotherapy for Rectal Cancer (clin trials gov NCT03426397) and was approved by the institutional review board of our institution. Patients with a complete response after CRTx are included in the study. Patients with a (near) complete response received a repeated response assessment, and all other patients undergo immediate TME surgery (Table S1). Patients who do not develop a clinical complete response after repeated assessments and patients who developed a regrowth later in the follow up undergo delayed TME surgery.

### 2.2. Patient Selection

Patients who received CRTx and TME surgery for adenocarcinoma of the rectum from January 2015–May 2020 were included. Exclusion criteria were delaying surgery for other reasons than regrowth, synchronous metastases, palliative treatment, other malignancy for which active treatment or surveillance. Patients with a local excision as treatment after CRTx were excluded, unless they were followed by a completion TME. Patients who received follow-up elsewhere or were lost to follow-up were excluded. Response assessment was performed 6–8 weeks after the end of CRTx with digital rectal examination, CT-chest and abdomen, pelvic MRI, and endoscopy. Patients with a clinical complete response entered the surveillance program with a three monthly MRI and endoscopy in the first two years as part of the W&W protocol. Patients with a near complete response were restaged after 6 weeks with endoscopy (near complete) and were at that time either included in the W&W protocol, or underwent delayed TME surgery.

### 2.3. Questionnaires

Quality of life was measured by the cancer-specific QLQ-C30 version 3.0 and the colorectal cancer-specific QLQ-CR29.The QLQ-C30 consists of five functional scales, three symptom scales and 6 single items. The 29-item QLQ-CR29 represents an update of the QLQ-CR38. The adapted Dutch version consists out of 4 scales and 17 single items [16].

The Cancer Worry Scale (CWS) is a validated questionnaire which consists out of four questions evaluating patients’ worry for cancer recurrence [17]. The validated Decision Regret Scale (DRS) consists out of 5 questions which assesses regret for a treatment decision [18]. The DRS was used to assess potential regret for choosing a W&W protocol in those patients requiring delayed surgery. The QLQ and CWS questionnaires were distributed by post in June 2020. Non-responders were contacted once by telephone after two weeks. The DRS was obtained by telephone, only in patients who underwent delayed surgery and who had returned the initial questionnaires.

### 2.4. Statistical Analysis

Statistical analyses were performed using IBM Statistical Package for the Social Sciences (SPSS version 23.0, Armonk, New York, NY, USA). Patient demographics, number of clinic visits from first presentation until surgery and peri-operative details were obtained from chart review. Follow-up was calculated from the end of CRTx. Scores and missing data of the EORTC questionnaires were handled according to the scoring manual. Higher functional scores indicated increased function, while higher symptom scores represent more severe symptoms. The CWS consists of 4 questions each with a 10-point Likert scale giving a maximum total score of 40. The DRS consists of 5 questions each with a 5-point Likert scale. Scores were handled according to the scoring manual, leading to a percentual score per question. Scores vary between 0–100, where a score >50 signifies a patient having decision regret. All scores were presented as means. To negate the effect of direct postoperative recovery, only questionnaires from patients at least six months postoperatively were included. Wilcoxon rank sum test, Fisher’s exact test, linear-by-linear association and general linear models were used to test for differences between groups.

It has been debated how well the QLQ-C30 performs in rectal cancer patients, specifically [19]. The Dutch validation study of the QLQ-CR29 suggested a modification of the original bowel symptom scores, leading to a new subscale with improved scale reliability for Dutch colorectal cancer patients [16]. For these reasons, we performed an exploratory factor analysis (EFA) to expose the latent factors in our dataset which we compared to the QLQ-C30 and CR29 questionnaires. An EFA based on eigenvalues (>1.0) using a Varimax rotation was used. In order not to overestimate effects, an appropriate loading factor of 0.75 was chosen based on our sample size of 51 [20]. Internal consistency was assessed by Cronbach’s alpha. Construct validity was tested through a ‘known groups comparison’; we hypothesized based on literature that patients undergoing APR would have a lower physical functioning score, lower body image, more loss of appetite and more sexual difficulty for men [21,22].

## 3. Results

### 3.1. Patient Demographics & Non-Responder Analysis

**Figure 1:**
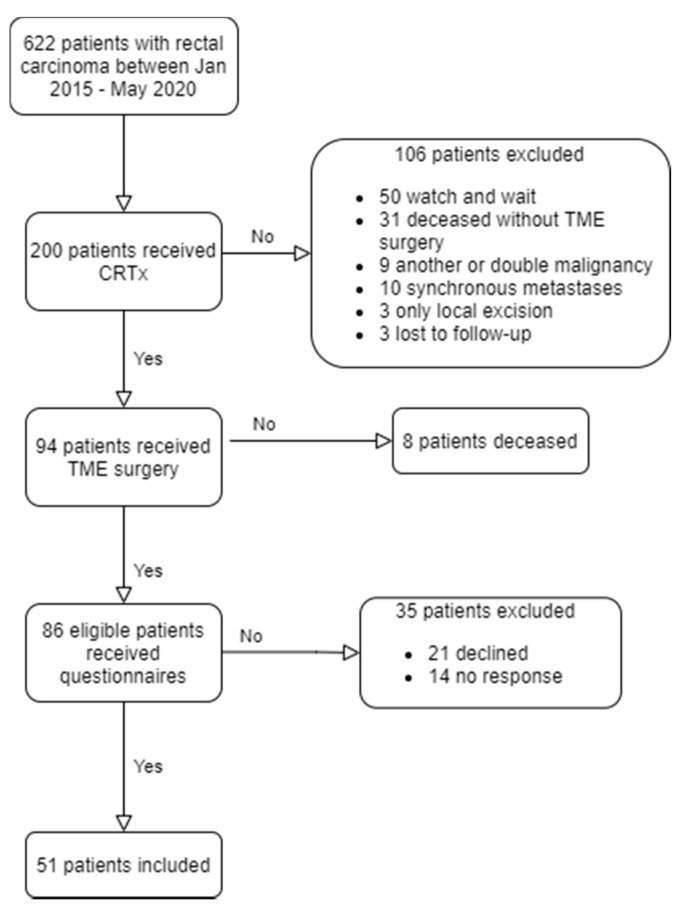
Inclusion strategy.

Between 2015 and 2020, 94 patients underwent either immediate or delayed TME surgery after CRTx. Eighty-seven patients were eligible for inclusion and were sent the questionnaires by post. Finally, 51 (59%) patients returned three full questionnaire after one follow-up call (Figure 1), 33 who had immediate TME and 18 who had delayed TME. Sixteen out of 18 patients returned the DRS questionnaire. Non-responder analysis showed no differences with responders, except for a trend towards more surgical reinterventions in the non-responder group (*p* = 0.06). Five out of these six non-responders underwent immediate surgery (Table S4). There were no significant differences in age, sex, ASA score, cTNM classification, laparoscopy, conversion, readmission, distant relapse and follow up between the immediate and delayed surgery groups (Table 1). The delayed surgery group had significantly more distal tumors (*p* = 0.03), more APR procedures (*p* = 0.02) and more ostomies at time of analysis (*p* = 0.02). Patients in the delayed surgery group had more clinic visits (5 vs. 2, *p* < 0.01).

### 3.2. QLQC30 & CR29

**Figure 2:**
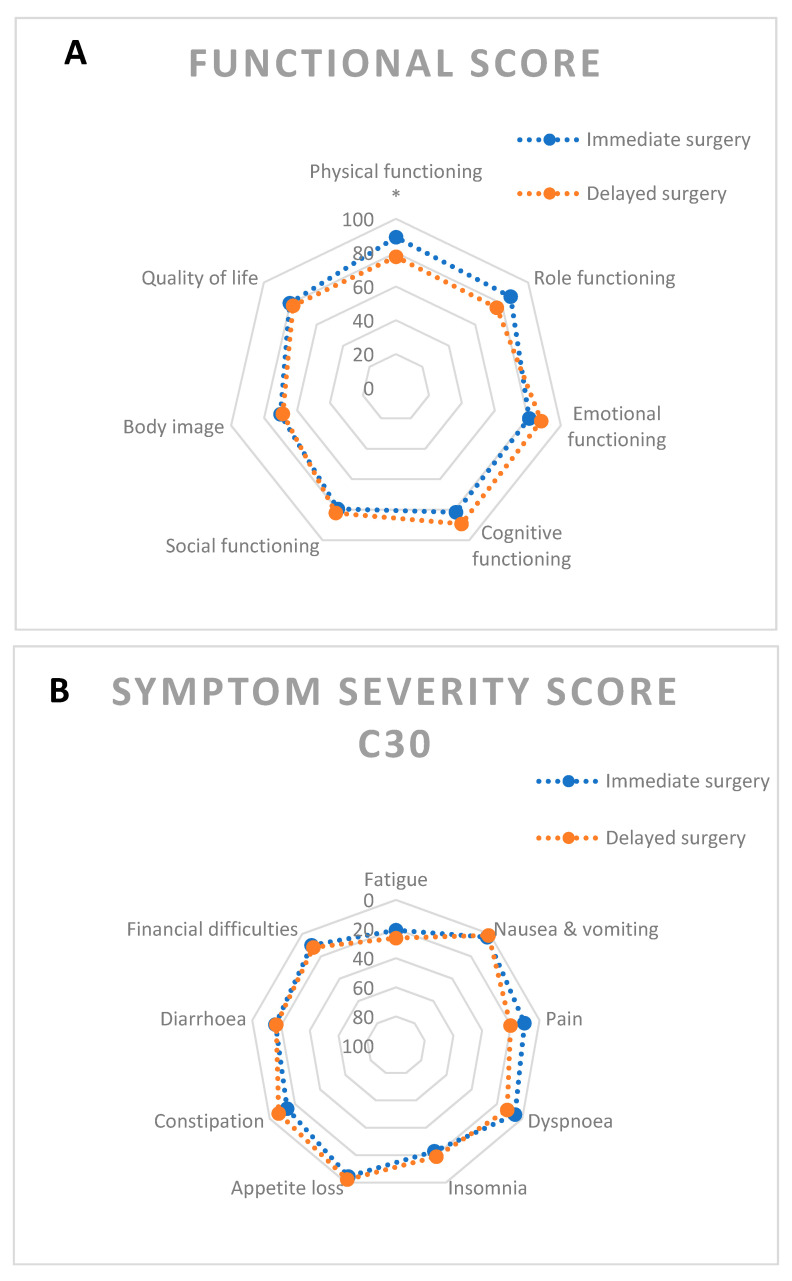

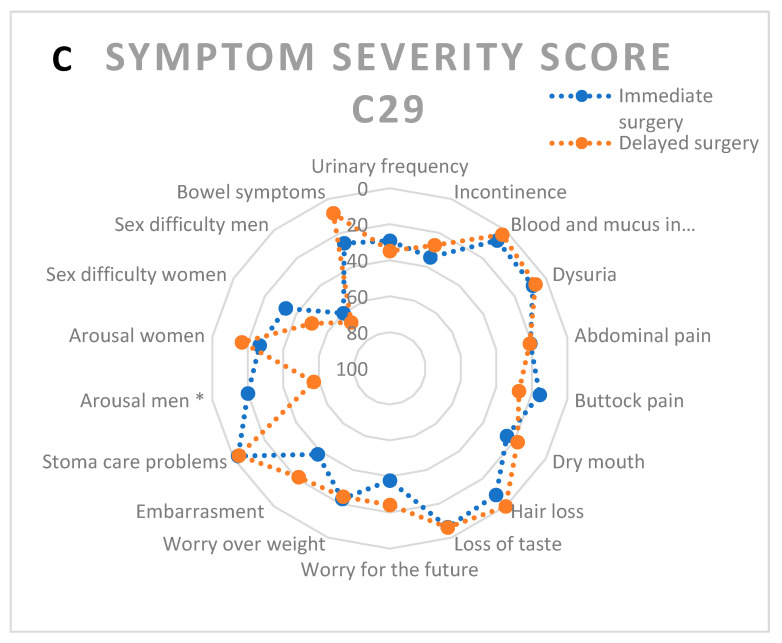
Radar charts depicting quality of life for immediate vs. delayed surgery groups. Means are given. * signifies statistical significance at *p* < 0.05. (**A**) Functional scores and QoL item. (**B**) QLQ-C30 symptom scores. (**C**) QLQ-CR29 symptom scores.

We observed a higher mean physical functioning score (89.2 vs. 77.6, *p* = 0.03) in the immediate surgery group. When corrected for operation type, no significant difference in mean physical functioning was found (*p* = 0.25). A non-significant lower mean role functioning score (86.7 vs. 76.2, *p* = 0.33) was found for the delayed surgery group. The QoL item had a similar mean score (80.5 vs. 78.0, *p* = 0.52). Arousal for men scored higher in the delayed surgery group (20.0 vs. 57.1, *p* = 0.02). There was no significant difference between surgical groups in the other function scales. All function and symptom scores are depicted in Figure 2. Tables S2 and S3 show scores for all scales and items.

### 3.3. Known Groups Comparison: APR vs. LAR

**Figure 3:**
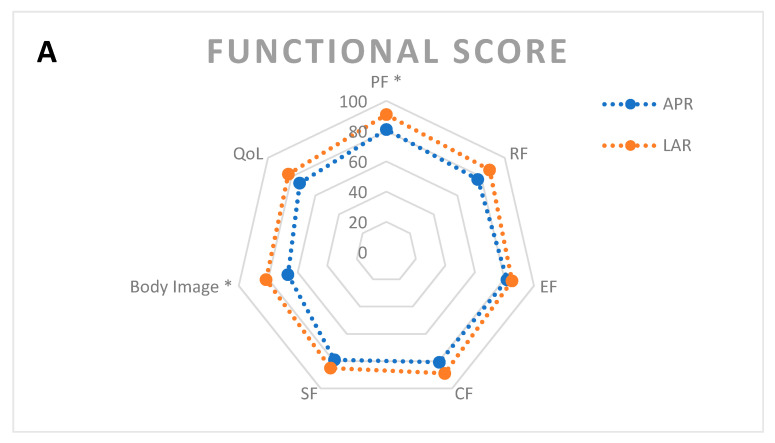

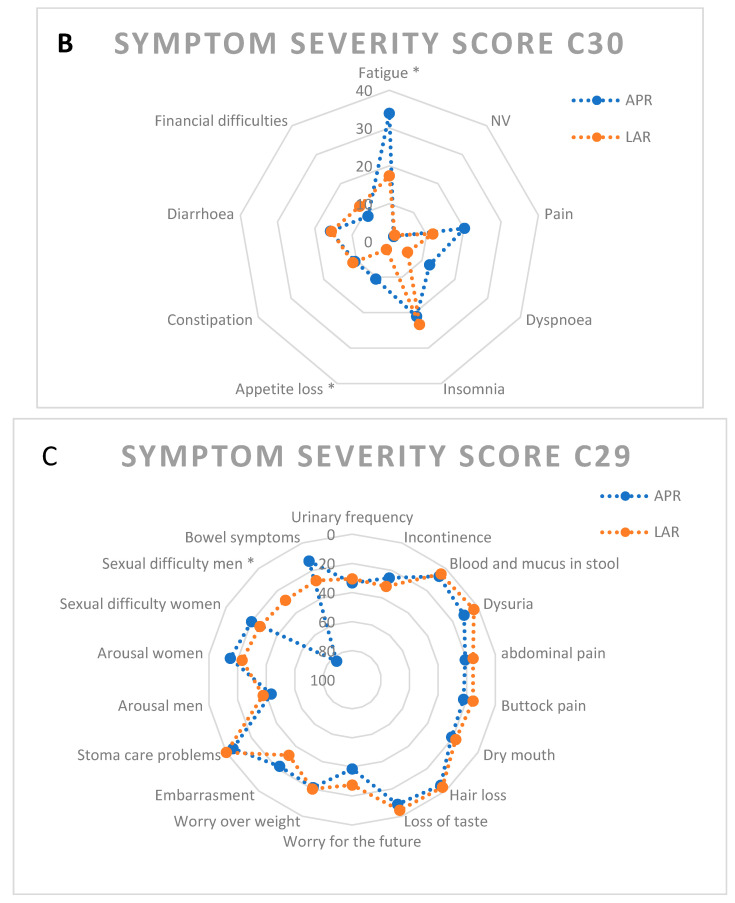
Radar charts depicting quality of life per operation type. Means are given. * signifies statistical significance at *p* < 0.05. (**A**) Functional scores and QoL item. (**B**) QLQ-C30 symptom scores. (**C**) QLQ-CR29 symptom scores.

Mean physical function scale score was lower in patients who underwent APR instead of LAR (77.5 vs. 89.5, *p* < 0.01). No difference was seen between LAR with a deviating stoma and APR for any of the subscales (*p* > 0.2). The APR group had a lower mean body image (66.7 vs. 83.3, *p* = 0.03). Mean symptom score for appetite loss was higher in the APR group (10.5 vs. 2.4, *p* = 0.047). Men in the APR group had a higher mean score for the sexual difficulty symptom item (83.3 vs. 28.6, *p* < 0.01). All scores are shown in Figure 3.

### 3.4. Cancer Worry Scale

The average CWS score was 14.0 in the immediate surgery group and 10.8 in the delayed surgery group (Table 2, *p* = 0.21).

### 3.5. Decision Regret Scale

Mean item scores varied between 10.7% and 16.1% between items (Table 3). No patient exhibited decision regret for any of the items.

### 3.6. Factor Analysis and Reliability

Exploratory factor analysis revealed six factors within the QLQ C30 explaining 76% of variance, of which the social functioning (Cronbach’s α = 0.82) and emotional functioning scale (α = 0.89) were reproduced with good internal consistency. The physical functioning scale (α = 0.79) without ‘ADL item’ no 5 and the role functioning scale were reproduced together as one factor (α = 0.92). All remaining factors did not form interpretable scales with reliabilities below 0.5.

Factor analysis also revealed six factors within the QLQ CR29 explaining 70% of variance, of which the body image scale (α = 0.85), urinary frequency scale (α = 0.67) and the blood and mucus in stool scale were reproduced (α = 0.50, originally α = 0.56) [16]. As in the original Dutch validation study, the original stool frequency scale (α = 0.61) showed greater internal consistency when added to a larger factor including all bowel and stoma problems (items 49–54, α = 0,87) [16]. All remaining factors did not form interpretable scales with reliabilities below 0.7. The CWS revealed one underlying factor explaining 70% of variance. Excellent scale reliability was found for the CWS and DRS (α = 0.86 and 0.84, respectively).

## 4. Discussion

Patients who undergo delayed TME surgery after CRTX have no impairment of quality of life or more worry for cancer than patients who undergo immediate TME surgery. These patients also exhibit little or no regret of the decision to enter a Watch & Wait protocol. From a quality of life perspective, it seems therefore that a repeated assessment strategy for near complete responders to identify all candidates for a W&W/NOM is not harmful.

QLQ-C30 scores in both groups are in the same range, and comparable to the normal population [23]. Indeed, most studies have found only limited differences between rectal cancer patients and the general population in terms of QoL [24]. It is believed that the experience of going through major surgery and insecurity about cancer, reshapes the patients’ perception of life in a positive way resulting in better reported QoL [25]. This so-called ‘post traumatic growth’ is well documented in (colorectal) cancer patients [26].

The delayed surgery group does not have a higher score on the CWS, with even a non-significant trend for a lower score (10.8 vs. 14.0, *p* = 0.2). A cut-off score of 14 on the CWS has been proposed in breast- and colorectal cancer patients to detect high fear of cancer [27] Patients with an excellent response to CRTx were told to have a favorable

prognosis, in addition to he possibility of treatment without surgery. Although eventually requiring a resection, patients might still feel they have a more favorable prognosis. Additionally, patients receiving delayed surgery have had significantly more outpatient clinic visits and examinations. The fact that these patients are in a prospective W&W study with additional counselling and attention, could have resulted in a greater sense of security and less worry for cancer [28].

Finally, we examined in those patients who underwent delayed surgery if they experienced regret towards the decision to participate. Probably, this is the most discerning indicator from a quality of life perspective. Even though these patients had to undergo delayed surgery, no patients showed decision regret with very low regret scores on all items.

The exploratory factor analysis reproduced most subscales in in the QLQ C-30 and CR-29. The physical functioning scale was reproduced with good internal consistency without the ‘ADL’ item, similar to a previous validation study [29]. The physical and role functioning scale were reproduced as one factor, suggesting that these questions answered a similar underlying ‘functioning parameter’ in our subset of patients. Equivalent to the original Dutch validation study, we found moderate scale reliability in the blood and mucus scale and greater internal consistency when all bowel and stoma items were combined in one scale (items 49–54) [16]. As reported earlier, our known groups comparison compared well to literature showing good construct validity. Summarizing, the QLQ-CR30 and CR29 showed good validity and was therefore feasible in our sample of neoadjuvant treated rectal cancer patients.

The main limitation of this study is the small sample size. In our watch & wait cohort, only 29 patients required delayed surgery and not all patients participated in the study. Further limitations are the presence of potential confounders and the 59% response rate. There is a higher proportion of patients receiving APR and having a stoma in the delayed surgery group. This could contribute to the non-significant lower mean physical functioning and role functioning score in the delayed surgery group. Patients with a stoma report lower scores on most QoL domains [30]. Moreover, Qol is reportedly higher in patients after LAR compared to APR, although not consistently. The present study showed significantly worse physical functioning, body image, appetite loss and male sexual difficulty after an APR than after a LAR.

The response rate in the present study was 59% and therefore selective non-response might have occurred. Although 59% is below average in surgical postal surveys, surveys in colorectal cancer patients often achieve 50–60% response rates [31]. An RCT in a cohort of 1200 cancer patients investigating response rates showed that the 55% response rate in colorectal cancer is lower than patients with prostate or breast cancer, even after correction for age, sex, marital status, and cancer stage [32]. Our non-responder analysis showed no differences between groups, except for a trend towards more surgical reinterventions in the non-responder group. Furthermore, the temporal variability between date of surgery and completion of the questionnaire is a limitation of this study.

## 5. Conclusion 

In conclusion, there is no impairment of quality of life or more worry for cancer in patients undergoing delayed TME surgery, as compared to immediate TME surgery. Therefore, this study supports a repeated response assessment strategy after CRTx for rectal carcinoma to identify all complete responders from a quality of life perspective.

## Figures and Tables

**Table 1:** Patient demographics immediate vs. delayed surgery groups. *p*-values in bold are significant (<0.05).

	Immediate Surgery N = 33		Delayed Surgery N = 18		*p*
	N (%)	Mean %	N (%)	Mean	
Age		63.9		61.2	0.33
Sex					0.57
M	17 (51.5%)		11 (61.1%)		
F	16 (48.5%)		7 (38.9%)		
ASAscore					0.74
1	11 (33.3%)		7 (38.9%)		
2	20 (60.6%)		9 (50.0%)		
3	2 (6.1%)		2 (11.1%)		
cT					0.54
3	30 (90.9%)		17 (100%)		
4	3 (9.1%)		0 (0%)		
cN					**0.04**
0	1 (3.0%)		4 (22.2%)		
1	9 (27.3%)		6 (33.3%)		
2	23 (69.7%)		8 (44.4%)		
CRTx interrupted					0.23
0	30 (90.9%)		14 (77.8%)		
1	3 (9.1%)		4 (22.2%)		
Endoscopic distance (cm)		10		6	**0.03**
Time to surgery (weeks)		15		35	**<0.01**
Type of operation					**0.02**
LAR	24 (72.7%)		7 (38.9%)		
APR	9 (27.30%)		11 (61.1%)		
Stoma-free survival					**0.02**
no stoma	21 (63.6%)		5 (27.8%)		
stoma in situ	12 (36.4%)		13 (72.2%)		
Laparoscopy					0.13
no	8 (24.2%)		1 (5.6%)		
yes	25 (75.8%)		17 (94.4%)		
Conversion					0.53
no	28 (84.8%)		17 (94.4%)		
yes	4 (12.1%)		1 (5.6%)		
unknown	1 (3.1%)		0 (0%)		
Readmission					0.23
no	23 (76.7%)		17 (94.4%)		
yes	7 (23.3%)		1 (5.6%)		
Distant relapse					0.46
no	26 (78.8%)		16 (88.9%)		
yes	7 (21.2%)		2 (11.1%)		
Follow up (months)		35		25	0.07

**Table 2:** CWS score for immediate vs. delayed surgery groups. Mean + SD are given. Score of >14 indicates high fear of recurrence.

	Immediate surgery		Delayed surgery	
	Mean	SD	Mean	SD
**CWS**	14.00	9.12	10.79	7.91

**Table 3:** DRS score for delayed surgery group. Score > 50 indicates decision regret.

Decision Regret Scale Score (n = 16)		
Item No.	Question	Score (%)
1	It was the right decision	12.5
2	I regret the choice that was made	10.7
3	I would go for the same choice if I had to do it over again	14.3
4	The choice did me a lot of harm	14.3
5	The decision was a wise one	16.1

## Data Availability

Study data is available for review, upon reasonable request.

## Supplementary Materials

The following are available online at https://www.mdpi.com/2072-6694/13/4/742/s1, Table S1: Definition of clinical complete response (cCR) and near cCR. Table S2: QLQ-C30 scores. Table S3 QLQ-CR29 scores. Table S4: Non responder comparison.
