# Supplementary material for: Delayed Surgery after Neoadjuvant Treatment for Rectal Cancer Does Not Lead to Impaired Quality of Life, Worry for Cancer, or Regret
Source: Cancers (Basel). 2021 Feb 11;13(4):742. doi: 10.3390/cancers13040742 (PMC7916848; doi:10.3390/cancers13040742)
Supplement: Supplementary file 1 [file cancers-13-00742-s001.pdf]

cancers

MDPI

Supplementary Materials: Delayed Surgery after Neoadjuvant Treatment for Rectal Cancer Does Not Lead to Impaired Quality of Life, Worry for Cancer, or Regret

Table S1. Definition of clinical complete response (cCR) and near cCR.

	Complete Response (cCR)	Near cCR
DRE	No palpable tumor	Superficial soft irregular tumor palpable
Endoscopy	White scar with teleangiectasia	○ Small persistent ulcus<br>○ Irregular thickening of mucosa and/or dysplasia at histology
MRI	○ Absence of tumor at T2W-MRI<br>○ Low signal at tumor location at b1000 DWI-MRI<br>○ Absence of suspicious lymph nodes at T2W-MRI.	○ Downstaging of the tumor with or without fibrosis. but with a heterogeneous or irregular aspect on MRI and/or<br>○ Limited focal area with high signal at b1000 DWI-MRI

Table S2. QLQ-C30 scores (mean + SD) for direct vs delayed surgery groups. P- values in **bold** are significant (< 0.05).

	Direct to Surgery		Delayed Surgery		
	Mean	SD	Mean	SD	*p*
PF	89.20	16.11	77.62	21.34	**0.03**
RF	86.67	22.49	76.19	29.75	0.33
EF	80.83	24.77	88.10	23.51	0.29
CF	81.67	24.11	89.29	16.80	0.66
SF	79.44	28.93	82.14	17.86	0.97
Fatigue	20.74	21.19	26.19	28.11	0.27
NV	2.78	7.69	1.19	4.45	0.58
Pain	10.56	18.82	20.24	27.09	0.34
Dyspnoea	5.75	12.81	11.90	28.06	0.84
Insomnia	22.99	29.69	19.05	31.25	0.63
Appetite loss	4.60	11.70	2.38	8.91	0.44
Constipation	13.79	24.43	7.14	14.19	0.61
Diarrhoea	16.09	26.16	16.67	28.50	0.89
Financial difficulties	10.00	19.87	11.90	24.83	0.78
Quality of life	80.46	22.52	77.98	22.31	0.53

Table S3. QLQ-CR29 scores (mean + SD) for direct vs delayed surgery groups. P- values in **bold** are significant (<0.05).

	Direct to surgery		Delayed surgery		
	Mean	SD	Mean	SD	*p*
**Urinary frequency**	29.17	29.5	34.85	30.23	0.97
**Body image**	70.14	26.52	68.69	33.27	0.20
**Incontinence**	34.37	29.48	27.27	15.41	0.50
**Blood and mucus in stool**	7.29	14.87	3.03	6.74	0.83
**Dysuria**	8.33	19.25	6.67	14.05	1.0
**Abdominal pain**	20.83	26.87	21.21	22.47	0.82
**Buttock pain**	15.56	30.52	27.27	35.96	0.07
**Dry mouth**	25	22.77	18.18	34.52	0.24

**Hair loss**	8.33	19.25	0	0	0.15
**Loss of taste**	6.25	18.13	6.06	20.1	0.38
**Worry for the future**	37.78	30.52	24.24	21.56	0.47
**Worry over weight**	22.92	26.44	24.24	36.79	0.95
**Embarrasment**	37.78	37.52	21.21	26.97	0.63
**Stoma care problems**	2.78	9.62	3.33	10.54	1.0
**Arousal men**	20	29.81	57.14	16.27	**0.02**
**Arousal women**	26.67	26.29	16.67	33.33	0.12
**Sex difficulty women**	33.33	25.2	50	70.71	0.63
**Sex difficulty men**	60	43.46	66.67	47.14	0.09
**Bowel symptoms**	26.04	25.07	8.33	16.2	0.07
**Bloating**	25	25.82	18.18	22.92	0.59

Table S4. Non responder comparison. One patient had a multivisceral resection classified as 'other'. There are no significant differences ($p < 0.05$) between groups.

	**Non responder**		**Responder**		
	**N (%)**	**Mean**	**N (%)**	**Mean**	***p***
Age		65.8		63.0	0.35
Sex					
M	23 (63.9%)		28 (54.9%)		0.40
F	13 (36.1%)		23 (45.1%)		
ASAscore					
1	11 (30.6%)		18 (35.3%)		0.59
2	24 (66.7%)		29 (56.9%)		
3	1 (2.8%)		4 (7.8%)		
Surgery					
Direct	25 (69.4%)		33 (64.7%)		0.65
Delayed	11 (30.6%)		18 (35.3%)		
Endoscopic distance from anal verge		8		8	0.61
cT					
2	1 (2.8%)		0 (0%)		0.41
3	30 (83.3%)		48 (94.1%)		
4	5 (13.9%)		3 (5.9%)		
cN					
0	3 (8.3%)		5 (9.8%)		0.71
1	13 (36.1%)		15 (29.4%)		
2	20 (55.6%)		31 (60.8%)		
MRF					
-	9 (25.0%)		8 (15.7%)		0.28
+	27 (75.0%)		43 (84.3%)		
Time to surgery (weeks)		27.3		22.6	0.35
Type of operation					
APR	14 (38.9%)		20 (39.2%)		0.97
LAR	21 (58.3%)		31 (60.8%)		
Other	1 (2.8%)		0 (0%)		
Laparoscopy					
no	7 (19.6%)		9 (17.6%)		0.84
yes	29 (80.6%)		42 (82.4%)		
Conversion					
no	34 (94.4%)		45 (88.2%)		
yes	2 (5.6%)		5 (9.8%)		0.40
unknown	0 (0.0%)		1 (2.0%)		
Surgical reintervention					0.06
no					

yes	28 (77.7%)	49 (95.7%)	
unknown	6 (16.7%)	2 (4.3%)	
	2 (5.6%)		
Readmission			
no	25 (75.8%)	43 (84.3%)	0.10
yes	8 (24.2%)	8 (15.7%)	
Distant relapse			
no	31 (86.1%)	41 (82.0%)	0.77
yes	5 (13.9%)	9 (18.0%)	
Follow up (months)	27.6	31.1	0.38
